# Efficacy and Safety of Yokukansan in Treatment-Resistant Schizophrenia: A Randomized, Multicenter, Double-Blind, Placebo-Controlled Trial

**DOI:** 10.1155/2015/201592

**Published:** 2015-04-14

**Authors:** Tsuyoshi Miyaoka, Motohide Furuya, Jun Horiguchi, Rei Wake, Sadayuki Hashioka, Masaya Thoyama, Kenta Murotani, Norio Mori, Yoshio Minabe, Masaomi Iyo, Shuichi Ueno, Sachiko Ezoe, Syuzo Hoshino, Haruo Seno

**Affiliations:** ^1^Department of Psychiatry, Shimane University School of Medicine, Izumo, Japan; ^2^Research Institute of Traditional Asian Medicine, Kinki University, Higashi-Osaka, Japan; ^3^Center for Advance Medicine and Clinical Research, Nagoya University Hospital, Nagoya, Japan; ^4^Psychiatry & Neurology, Hamamatsu University School of Medicine, Hamamatsu, Japan; ^5^Psychiatry, Kanazawa University School of Medicine, Kanazawa, Japan; ^6^Psychiatry, Chiba University School of Medicine, Chiba, Japan; ^7^Psychiatry, Ehime University School of Medicine, Matsuyama, Japan; ^8^Medical Center for Translational Research, Osaka University Hospital, Suita, Japan; ^9^Psychiatry, Takeda General Hospital, Fukushima, Japan; ^10^Psychiatry, Matsue Aoba Hospital, Matsue, Japan

## Abstract

*Objectives*. We aimed at evaluating both the efficacy and safety of TJ-54 (Yokukansan) in patients with treatment-resistant schizophrenia. This randomized, multicenter, double-blind, placebo-controlled study was conducted. *Methods*. One hundred and twenty antipsychotic-treated inpatients were included. Patients were randomized to adjuvant treatment with TJ-54 or placebo. During a 4-week follow-up, psychopathology was assessed using the Positive and Negative Syndrome Scale (PANSS). *Results*. TJ-54 showed a tendency of being superior to placebo in reduction total, positive, and general PANSS scores in treatment-resistant schizophrenia, but the difference was not statistically significant in both per-protocol set (PPS) and intention-to-treat (ITT). However, in PPS analysis, compared to the placebo group, the TJ-54 group showed statistically significant improvements in the individual PANSS subscale scores for lack of spontaneity and flow of conversation (TJ-54: −0.23 ± 0.08; placebo: −0.03 ± 0.08, *P* < 0.018), tension (TJ-54: −0.42 ± 0.09; placebo: −0.18 ± 0.09, *P* < 0.045), and poor impulse control (TJ-54: −0.39 ± 0.10; placebo: −0.07 ± 0.10, *P* < 0.037). *Conclusions*. The results of the present study indicate that TJ-54 showed a tendency of being superior to placebo in reduction PANSS scores in treatment-resistant schizophrenia, but the difference was not statistically significant. However, compared to the placebo group, TJ-54 group showed statistically significant improvements in the individual PANSS subscale scores.

## 1. Introduction

Treating schizophrenia patients who either respond minimally to treatment or do not respond at all represents a major clinical challenge. Patients resistant to antipsychotic treatment constitute up to 20–25% of all patients with schizophrenia [1]. Despite the introduction of atypical antipsychotics for treatment-resistant schizophrenia, clinicians are faced with the challenge of treating drug-resistant patients. A majority of these individuals experience negative symptoms that account for a significant impact on morbidity and diminished quality of life associated with chronic forms of schizophrenia. In such situations, alternative therapeutic approaches involving a combination of standard antipsychotic treatment with other drugs are employed. However, only a handful of adjunctive treatment options have been systematically studied in clinical settings. Adjunctive therapies can result in polypharmacy and, therefore, require careful monitoring of any of its associated benefits.

Yokukansan (TJ-54) was developed in 1555 as a remedy for restlessness and agitation in children [2]. Prompted by the increasing life expectancy of the Japanese population, geriatricians have begun to use this traditional treatment for behavioral and psychological symptoms of dementia (BPSD) in the elderly. For example, Iwasaki et al. reported successful TJ-54 treatment of 2 patients with BPSD in an extended care unit [3, 4]. Moreover, in a randomized, observer-blind, controlled trial, TJ-54 improved BPSD and activities of daily life [5]. Furthermore, in a previous open-label study, we reported TJ-54 therapy to be an effective and a well-tolerated option for several neuropsychiatric disorders, namely, borderline personality disorder, drug induced tardive dyskinesia, treatment-resistant schizophrenia, late-onset schizophrenia, visual hallucinations due to vision loss, and pervasive developmental disorder [6–14]. Clinical trials and detailed case reports of TJ-54 [15–25] suggest the its possibility of representing a new treatment approach for treatment-resistant schizophrenia. TJ-54 extract is a herbal medicine containing the rhizome of* Atractylodes lancea* (Japanese Pharmacopoeia: JP), sclerotium of* Poria* (JP),* Cnidium rhizome* (JP), hook of* Uncaria* (JP), root of Japanese angelica (JP), root of* Bupleurum* (JP), and* Glycyrrhiza* (JP). TJ-54 has been used for the treatment of insomnia, irritability, and convulsions for many years, and the pharmacological effects of TJ-54 are thought to be associated with 5-hydroxytryptamine (5-HT) receptors [3, 5].

This study aimed to evaluate the efficacy and tolerability of TJ-54 as an add-on pharmacotherapy for clinical symptoms in patients with treatment-resistant schizophrenia over a 4-week period. We hypothesized that TJ-54 would reduce some symptoms of treatment-schizophrenia consequently resulting in improved social and global functioning. To the best of our knowledge, no double-blind, placebo-controlled trials have been performed to examine the clinical efficacy of TJ-54 as an adjunctive to antipsychotics therapy for the treatment of treatment-resistant schizophrenia.

## 2. Materials and Methods

### 2.1. Materials

TJ-54 extract was provided by Tsumura & Co. (Tokyo, Japan). TJ-54 is a mixture of dried herbs containing 4 g of rhizome of* A. lancea*, 4 g of* Poria*, 3 g of C.* rhizome*, 3 g of* A. radix* (*A. acutiloba*), 2 g of* B. radix*, 1.5 g* G. radix*, and 3 g of* Uncariae uncis cum ramulus*. These herbs are registered in the JP (ver. 15). The amount of active ingredients in the powder extract has been found to be equivalent to that in the standard solution of individual components as confirmed by thin-layer chromatography. Manufacturing processes and quality control measures were in conformance with Good Manufacturing Practices. TJ-54 has been approved by the Ministry of Health, Labour and Welfare, and covered by the National Health Insurance plan. The compounds on the chromatogram were classified on the basis of the constituent herbs of TJ-54 (Table 1) [23].

### 2.2. Study Participants

The study involved inpatients from 34 psychiatric hospitals in Japan. On the basis of data from previous reports, we calculated the required sample size to be 49 with an *α*-error of 0.05 and power of 0.9 [8, 26]. The protocol was approved by the Shimane University School of Medicine Subjects Review Committee and carried out in accordance with the Declaration of Helsinki and Tokyo for Humans, with written informed consent obtained from all recruited subjects.

Male or female hospitalized patients were eligible for inclusion in the study if they met all of the following criteria: age of 20–59 years; a primary Diagnostic and Statistical Manual of Mental Disorders, 4th edition, text revision (DSM-IV-TR) [27], diagnosis of schizophrenia, established by the Structured Clinical Interview for Diagnostic and Statistical Manual of Mental Disorders (SCID), Fourth Edition [28], with a length of at least 3 years; history of documented treatment-resistant status, which is defined as the absence of clinically significant improvement after treatment with at least 2 neuroleptics for 6 weeks or longer after receiving a full dose equivalent to 600 mg/day of chlorpromazine; presence of persistent positive symptoms as evidenced by a score of at least 10 on the positive symptom subscale of the Positive and Negative Syndrome Scale (PANSS) [29]; and an overall score of at least 60 on the PANSS and ≥4 (moderately ill) on the Clinical Global Impression-Severity (CGI-S) [30]. Treatment-resistant schizophrenia was defined as little or no response to treatment from at least two adequately dosed antipsychotic trials for at least 4 weeks including at least one second-generation antipsychotic (>600 mg/day of chlorpromazine equivalent), presence of persistent positive psychotic symptoms characterized by PANSS scores of 4 or higher on at least two items from the positive subscale, a PANSS total score >60, and CGI >4; constructs were adopted from the US multicenter trial of clozapine [31]. The current episode period is at least 6 weeks at the screening examination to exclude patients with acute phase. Exclusion criteria included pregnancy, lactation, other clinical significant or unstable conditions, and history of alcohol or substance abuse in the last 6 months. No patients had been treated by clozapine in this clinical trial. Because the clozapine has not been available in Japan until 2009, it might have been difficult to include patients who had gone clozapine trial in the past.

### 2.3. Study Design

**Figure 1:**
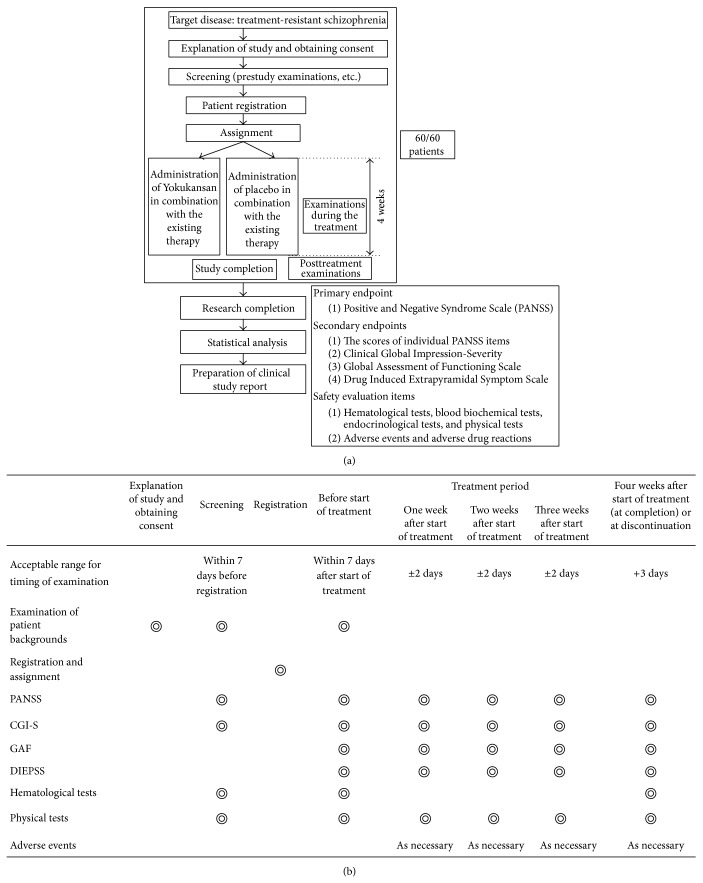
Study design (a) and schedule (b).

The study was a 4-week, double-blind, placebo-controlled, fixed-flexible dose trial (Figure 1(a)). Physical examination and routine laboratory test including evaluation of prolactin and plasma clozapine levels were carried out at baseline. Existing medications at baseline remained unchanged, whereas initiation of other psychotropic medications was not permitted during the trial. Patient status was reviewed every week by evaluation of clinical outcomes and vital signs (blood pressure and pulse rate) (Figure 1(b)). Subsequently, the dosettes were reviewed and medication was dispensed for the following week.

### 2.4. Sample Size

Based on our previous studies on effects of TJ-54 on PANSS positive symptoms of treatment-resistant schizophrenia [8, 9], we assumed a difference of 4 on PANSS negative symptom subscale, a standard deviation of 6, a two-side significance of 5%, and power of 90% for calculation of sample size. A sample size of 90 (each group, 45) was calculated, and, assuming a 25% attrition rate, a final sample size of 120 was achieved (each group, 60).

### 2.5. Randomization, Allocation Concealment, and Blinding

A computer random number generator was used for randomizing the patients in a 1 : 1 ratio (blocks of four). Treatment allocation was concealed from the study participants and the physicians who rated them using sequentially numbered, opaque, and sealed envelopes. Random allocation and clinical assessment of the participants were done by separate persons. The patients, the clinician who referred them, the psychiatrist who rated the participants and prescribed the medication, and the statistician were blind to allocation.

### 2.6. Medication

Subjects were randomized to receive either TJ-54 or placebo (1 : 1). The active drug and the placebo were constituted in a powder form, which was identified by a company specializing in the preparation of drug for experimental trials (Tsumura Co.).

Study participants in the test group received 2.5 g of TJ-54 powder three times for weeks. Study medication was dispensed in dosettes, and the drug package counts were determined at each visit to monitor adherence. Patients continued their regular antipsychotic treatment. Psychiatrists were requested to delay any changes in antipsychotic treatment until after the trial to keep the dose of underlying antipsychotics constant.

### 2.7. Clinical Measurements

Assessments included evaluation of clinical response and adverse effects. Clinical responsiveness was evaluated with the help of PANSS, CGI-S, and global assessment of functioning (GAF) [27]. Global adverse effects and movement disorder were evaluated for the assessing tolerability. Movement disorder was evaluated with the help of Drug Induced Extrapyramidal Symptom Scale (DIEPSS) [32]. Efficacy and tolerability scores were evaluated by the principal investigator.

### 2.8. Outcomes

The primary outcome measure was change in total, positive, negative, and general PANSS scores. Secondary outcomes were changes in individual PANSS scores and CGI-S.

### 2.9. Statistical Analysis

In this study, per-protocol set was a priori defined in the protocol, considering this trial is multicenter trial and TJ-54 is very unique herbal medicine. So, analyses were performed on a per-protocol set. If a patient dropped out, all outcome measures were assessed within a week. We first examined baseline differences between the randomized groups.

To assess the efficacy of TJ-54, we used linear mixed models for fixed and random effects with the dependent variable being the PANSS score. Time and group were entered as continuous and dichotomous independent variables, respectively. The 4-week treatment efficacy of TJ-54 compared to placebo for each outcome was estimated by entering a group-by-time interaction term. Mixed models are superior for the analysis of longitudinally correlated data with missing values [33]. These models make use of all available data and consequently have the best statistical power. Dependency of repeated assessments of the outcome scores was taken into account by including random effects for patients with an unstructured variance-covariance structure. Model-estimated marginal means for each follow-up visit were calculated according to treatment group. In addition, we performed a more conservative analysis of the PANSS scores with an intention-to-treat data set, defined as the subset of patients who completed baseline and at least one postbaseline evaluation, using a last-observation-carried-forward (LOCF) method for those who dropped out of the study during follow-up. In these analyses, differences in score changes between the randomized groups were evaluated using the unpaired *t*-test.

Analyses, blind to treatment status, were performed using the SAS statistical package (SAS Institute Inc., Cary, NC). The 2-sided level of significance was set at 0.05. Values are reported as mean with corresponding 95% confidence intervals (CIs) wherever appropriate.

## 3. Results

### 3.1. Subject Demography, Clinical Characteristics, and Follow-Up

**Figure 2:**
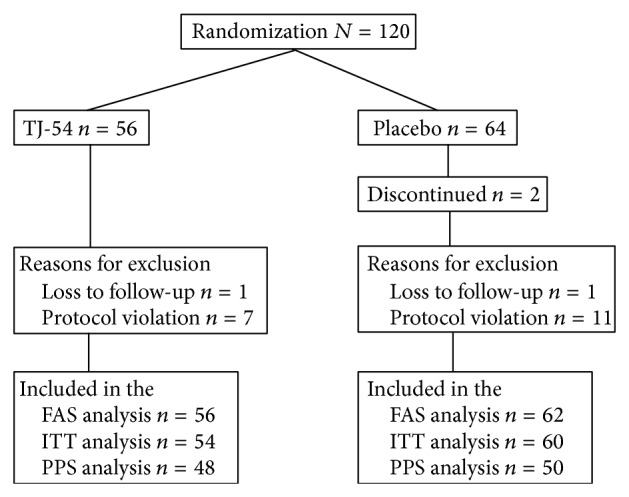
Study design and patient flow chart.

One hundred and twenty patients treated with antipsychotics were included in the run-in period (Figure 2). Of these 120 patients, 120 were eligible and were randomly assigned. Baseline characteristics are shown in Table 2. The percentage of the men in the placebo group was slightly higher than in the TJ-54 group; however, the difference was not significant. Other baseline characteristics showed no material differences. Notably, the scores of the total PANSS and PANSS subscales were comparable between the 2 randomized groups. Eight patients in the TJ-54 group and 14 in placebo group did not complete follow-up. In the placebo group, 3 patients lacked motivation and 11 violated the study protocol. In the TJ-54 group, 1 patient lacked motivation, while 7 violated the study protocol. The average compliance from randomization to the last follow-up was estimated by package count and was found to be 99% in the TJ-54 group and 100% in the placebo group. The patients were enrolled between May 2011 and August 2012. Follow-up ended in December 2012.

### 3.2. Psychopathology

**Figure 3:**
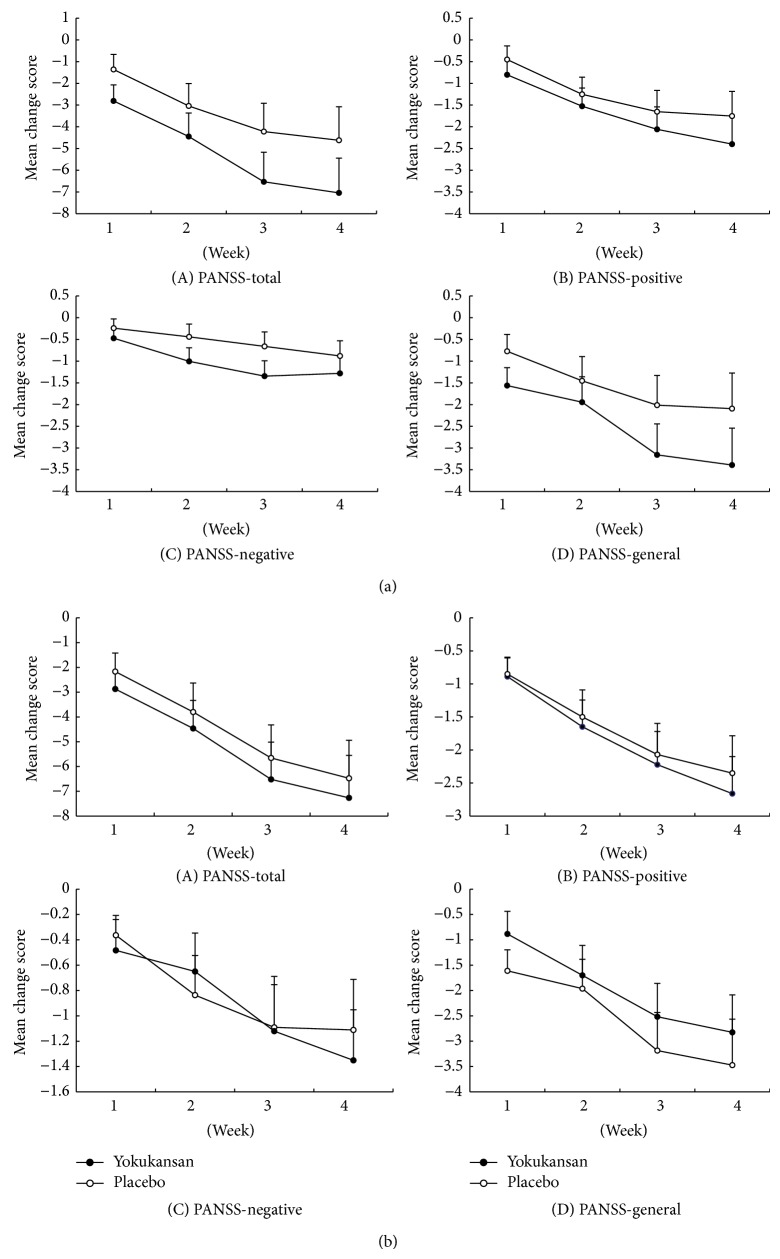
(a) Model-estimated marginal means of total and subscale scores of Positive and Negative Syndrome Scale (PANSS) relative to baseline values according to treatment group and follow-up visit (per-protocol set). (A) Total PANSS scores; (B) positive PANSS subscores; (C) negative PANSS subscores; and (D) general psychopathology scores. Error bars indicate standard error of the mean. (b) Model-estimated marginal means of total and subscale scores of Positive and Negative Syndrome Scale (PANSS) relative to baseline values according to treatment group and follow-up visit (intention-to-treat). (A) Total PANSS scores; (B) positive PANSS subscores; (C) negative PANSS subscores; and (D) general psychopathology scores. Error bars indicate standard error of the mean.

**Figure 4:**
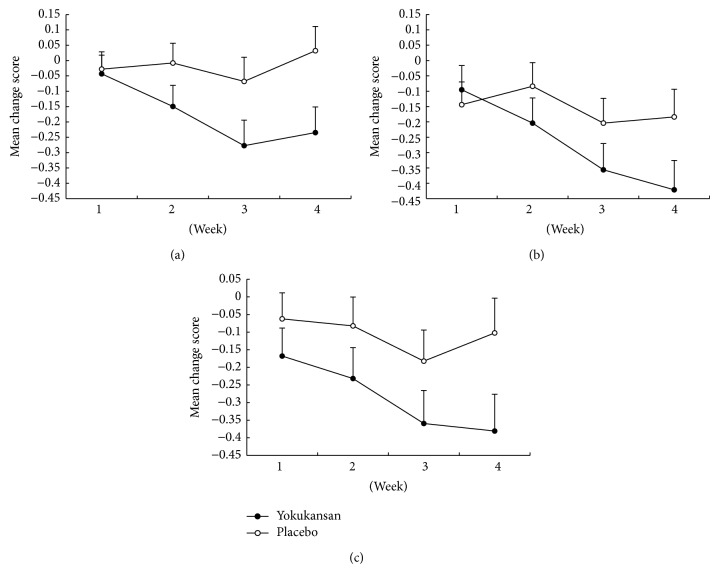
Model-estimated marginal means of individual score for Positive and Negative Syndrome Scale (PANSS). Lack of spontaneity and flow, tension, and poor impulsive control items: (a) lack of spontaneity and flow (item N6); (b) tension (item G4); (c) poor impulse control (item G14) scores. Error bars indicate standard error of the mean.

Group-specific model-estimated marginal means of the PANSS scores at each follow-up visit are shown in Figure 3. The figure shows that decreases in scores of the total and PANSS subscales with time were more pronounced in the TJ-54 group than in the placebo group, more so with respect to the general PANSS subscale scores.  Table 3 shows mean change in the PANSS scores from baseline to the last follow-up (LOCF) according to treatment group and estimates of treatment efficacy. Mixed models showed larger mean decreases in the TJ-54 group than in the placebo group; however, there was no statistical difference in the total PANSS score and PANSS subscales scores for positive symptoms, negative symptoms, and general symptoms between the groups. The group-specific model-estimated marginal means of the individual PANSS lack of spontaneity and flow conversation (N6), tension (G4), and poor impulse control (G14) subscores at each follow-up visit are shown in Figure 4. These 3 parameters improved significantly with TJ-54 compared to placebo, and the improvement in lack of spontaneity and flow conversation was more pronounced with TJ-54 (Table 3). Other psychopathological subscores did not vary between the groups (data not shown).

### 3.3. Safety and Tolerability

To establish safety and tolerability of TJ-54, full analysis set (FAS) testing was performed. Treatment with TJ-54 was well tolerated. Discontinuation from the study because of an adverse event was not observed (Figure 2; Table 4). There was no statistically significant time or group^  x^ time interaction effects for DIEPSS (Table 3). There were no clinically relevant changes from baseline in term of body weight, laboratory evaluations, vital signs, or QTc interval in either group.

### 3.4. The Response Rate of TJ-54

The response rates of TJ-54 and placebo were presented in 25% steps (Table 5). TJ-54 showed a tendency of superiority to placebo in the response rate, but the difference was not statistically significant.

## 4. Discussion

This is the first double-blind, placebo-controlled investigation of the therapeutic value of TJ-54 in the treatment of psychotic disorders. TJ-54 showed a tendency of being superior to placebo in reduction total, positive, and general PANSS scores in treatment-resistant schizophrenia, but the difference was not statistically significant. However, compared to the placebo group, TJ-54 group showed statistically significant improvements in the individual PANSS subscale scores for lack of spontaneity and flow of conversation, tension, and poor impulse control.

Mechanisms of TJ-54 action on psychiatric symptoms have been reported previously. TJ-54 inhibits 2,5-dimethoxy-4-iodoamphetamine-induced head-twitch response, decreases the expression of 5-HT2A receptors in the prefrontal cortex [34], possesses 5-HT1A partial agonistic effects [35], and has inhibitory effect on glutamate-mediated excitotoxicity [36, 37]. It can be thought that TJ-54 affects these neurotransmitters and receptors in a multifaceted manner. The herbal ingredients and their components have the following pharmacological effects, which may be responsible for the clinical effects seen with TJ-54. An aqueous extract of the hooks and stems of* U. sinensis* Havi.,* Uncariae uncis cum ramulus*, protected cultured cerebellar granule cells against glutamate-induced neuronal death [38]. Oxindole alkaloids, such as isorhynchophyline, isocorynoxetine, and rhynchophylline, and indole alkaloids such as hirsuteine and hirsutine are the active components of* Uncariae* [39]. Rhynchophylline and isorhynchophyline show antagonistic effects at the* N*-methyl-*D*-aspartame receptors [40]. Geissoschizine methyl ether (GM), corynantheine, and dihydrocorynantheine obtained from* Uncariae uncis cum ramulus* were found to be partial agonists for 5-HT receptors [41]. Glycyrrhizin, one of the main components of* G. radix*, and its metabolite, 18 beta-glycyrrhetinic acid, may be responsible for amelioration of dysfunction of glutamate transport in astrocytes [36]. Recently, an* in vitro* binding study demonstrated TJ-54 to be an agonist at the 5-HT1A and dopamine (DA) 2 receptors. Another* in vitro* experiment revealed that geissoschizine methyl ether (GM), a galenicals constituent of TJ-54, potently, with comparable affinity, binds to 5-HT1A and DA2 receptors [10, 39, 42].

TJ-54 at a dose of 7.5 g/day was associated with marked improvement in lack of spontaneity and flow of conversation, tension, and poor impulsive control. In light of research that suggested that the dysfunction of DA, 5-HT, and glutamate is associated with maladaptive behavior in schizophrenia, the unique mechanism of action of TJ-54 whereby it exerts partial D2 agonistic, 5-HT1A agonistic, and 5-HT2A and glutamate antagonistic effects [9, 43] may prove to be important for both its effectiveness and tolerability in treatment-resistant schizophrenia [44].

Although highly speculative, the positive effects on the 3 PANSS items may be due to TJ-54 being partial 5-HT1A agonist [10, 35]. A putative association has been hypothesized between partial agonism at 5-HT1A receptors and improvements in anxiety and depression, as well as the negative symptoms of schizophrenia [10].

When TJ-54 was combined with antipsychotics, the therapeutic benefits were significantly enhanced. Compared to the patients treated with placebo, the patients who received adjunctive TJ-54 therapy showed greater improvements in most efficacy measures, although the differences were not statistically significant. Lack of spontaneity and flow of conversation, tension, and poor impulse control scores on the PANSS were significantly different between the TJ-54 and the placebo groups. Both last-observation-carried-forward and observed case data analyses consistently demonstrated that the endpoint mean reduced scores of patients who received adjunctive TJ-54 therapy were approximately 2.4 points on the PANSS overall scale and about 0.5–1.3 points on the 3 subscales and were higher than the corresponding scores in the placebo group. The CGI-S scores did not differ significantly between the groups. These results suggest that TJ-54 is superior to placebo in augmenting the therapeutic effects of antipsychotics, particularly in improving lack of spontaneity and flow of conversation, tension, and poor impulsive control. Nevertheless, we found that, compared to placebo, TJ-54 when given with antipsychotics did not exert significantly different effects in items of improvement in PANSS positive, negative, and general symptom subscale scores. This result is inconsistent with those of previous open-label studies and case studies in which an apparent effect of TJ-54 as an add-on therapy was observed in reducing hallucinations and delusions [8]. One possible explanation for the inconsistency may be the differences in baseline clinical features of the study subjects. Unlike previous studies in which positive symptoms were the principal clinical manifestation, the present study involved patients with chronic schizophrenia who had dominant negative symptoms with significant cognitive disturbances compared to positive symptoms. The study results appear to suggest that TJ-54 plays a limited role in improving positive symptoms of chronic schizophrenia.

TJ-54 is generally well tolerated and has no major side effects [5]. On the other hand, there are reports suggesting that TJ-54 may cause nausea and/or hypokalemia in some elderly patients [15]. However, these side effects were not observed in any of the patients in this study. Overall, TJ-54 was well tolerated with no severe or serious adverse effects.

We should discuss the finding of no significant difference in PANSS positive or negative symptoms (was this due to response in placebo group or lack of efficacy of TJ-54 for these symptoms?). It is pity that the effects were basically limited to the symptoms of N6, G4, and G14 items and no clinically significant benefit was found for the other symptoms. Several study limitations should be considered. The 4-week treatment duration was too short, which might have prevented us from exploring the efficacy of TKS. Therefore, now, we are conducting 12-week trial.

The results of this study suggest that TJ-54 has the potential to be an effective and a well-tolerated treatment for improving lack of spontaneity and flow of conversation, tension, and poor impulse control in treatment-resistant schizophrenia.

## 5. Limitations

The main limitation of the present study is its short duration. Long-term desirable or untoward effects of TJ-54 might emerge later on, and, therefore, the optimal duration of this treatment remains to be determined. Another limitation was the small patient sample. Larger trials are needed to derive definitive conclusions, despite the coherent results of the present study, which are consistent with those previously reported.

## Figures and Tables

**Table 1:** Classification of the compounds identified in the three-dimensional chromatogram.

Constituent of TJ-54	Compounds
*Atractyloids lanceae rhizome *	4E,6E,12E-Tetradecatriene-8,10-diyne-1,3,14-triol
	12-Isovaleroyl-2E,8E,10E-triene-4,6-diyne-1,14-diol
	14-Isovaleroyl-2E,8E,10E-triene-4,6-diyne-1,12-diol, atractylodin

*Cnidii rhizoma *	Ferulic acid, ligustilide

*Uncariae uncis cum ramulus *	Geissoschizine methyl ether, hirsuterine, hirsutine

*Angelicae radix *(*Angelica acutiloba*)	Xanthotoxin, ligustilide

*Bupleuri radix *	Saikosaponin b1, saikosaponin b2

*Glycyrrhizae radix *	Formononetin, formononetin-7-*O*-glucoside
	Liquiritigenin, liquiritin, liquiritin apioside, glycyrrhizin, glycyroside, isoliquiritin apioside, isoliquiritin, isoliquiritigenin, glycylcoumarin

**Table 2:** Baseline characteristics.

	TJ-54 (*n* = 48)	Placebo (*n* = 50)	Test statics
Age (years)	46.4 ± 9.7	5.4 ± 9.6	ns
Gender (males/females)	28/20	32/18	ns
Duration of disease (years)	23.7 ± 10.4	22.5 ± 9.6	ns
Duration of treatment (years)	3.2 ± 10.5	21.0 ± 10.3	ns
CPZ equivalents (mg)	1905.8 ± 1541.5	1941.8 ± 1948.1	ns
Type			
Paranoid	39	35	
Catatonic	6	5	
Undifferentiated	3	10	ns
PANSS (total)	113.0 ± 23.0	111.1 ± 21.1	ns
PANSS (positive)	28.6 ± 5.4	28.7 ± 6.0	ns
PANSS (negative)	27.9 ± 7.1	27.8 ± 7.3	ns
PANSS (general)	56.6 ± 13.3	54.6 ± 11.9	ns
CGI-S	5.4 ± 0.8	5.5 ± 0.8	ns
GAF	27.4 ± 7.5	27.2 ± 6.4	ns
DIEPSS	4.3 ± 4.0	4.8 ± 4.5	ns

CPZ: chlorpromazine; PANSS: Positive and Negative Syndrome Scale; CGI-S: Clinical Global Impression-Severity; GAF: Global Assessment of Functioning; DIEPSS: Drug Induced Extrapyramidal Symptoms Scale.

**Table 3:** Changes in efficacy measures from baseline to week 4 as determined by mixed-model repeated-measurements analysis (per-protocol set and intention-to-treat).

	TJ-54	Placebo	*P*
	*n* = 48	*n* = 50	

PPS			
PANSS (total)	−7.04 ± 1.59	−4.62 ± 1.53	ns
PANSS (positive)	−2.40 ± 0.59	−1.75 ± 0.56	ns
PASS (negative)	−1.28 ± 0.37	−0.88 ± 0.35	ns
PANSS (general)	−3.39 ± 0.36	−2.09 ± 0.34	ns
PANSS (N6 item)	−0.23 ± 0.08	0.03 ± 0.08	<0.018^*^
PANSS (G4 item)	−0.42 ± 0.09	−0.18 ± 0.09	<0.045^*^
PASS (G14 item)	−0.39 ± 0.10	−0.07 ± 0.10	<0.037^*^
CGI-S	−0.36 ± 0.09	−0.25 ± 0.07	ns
GAF	2.94 ± 0.70	2.82 ± 0.86	ns
DIEPSS	−0.57 ± 0.21	−0.68 ± 0.19	ns

	*n* = 54	*n* = 60	

ITT			
PANSS (total)	−7.12 ± 1.70	−6.47 ± 1.54	ns
PANSS (positive)	−2.66 ± 0.56	−2.35 ± 0.57	ns
PASS (negative)	−1.29 ± 0.41	−1.13 ± 0.39	ns
PANSS (general)	−3.47 ± 0.91	−2.82 ± 0.39	ns
PANSS (N6 item)	−0.25 ± 0.09	−0.01 ± 0.09	<0.021^*^
PANSS (G4 item)	−0.43 ± 0.10	−0.22 ± 0.09	<0.048^*^
PASS (G14 item)	−0.42 ± 0.11	−0.15 ± 0.10	<0.028
CGI-S	−0.35 ± 0.09	−0.26 ± 0.08	ns
GAF	2.98 ± 0.95	2.63 ± 0.72	ns
DIEPSS	−0.54 ± 0.19	−0.57 ± 0.14	ns

Absolute values are given as scores at week 0 (baseline) minus those at week 4 (endpoint) (positive values indicate improvement). Data are not adjusted for baseline differences. ^*^Significantly different. PANSS: Positive and Negative Syndrome Scale; CGI-S: Clinical Global Impression-Severity; GAF: Global Assessment of Functioning; DIEPSS: Drug Induced Extrapyramidal Symptoms Scale. PPS: per-protocol set; ITT: intention-to-treat.

**Table 4:** Definite, probable, and possible adverse reactions of study intervention by week 4.

	TJ-54 (*n* = 56)	Placebo (*n* = 62)
Psychological	0	1
Neurological	0	0
Gastrointestinal	0	1
Genitourinary	0	0
Musculoskeletal	0	0
Dermatological	0	0
Respiratory	0	0
Cardiovascular	0	0
Infection	0	0
Ear, nose, and throat	0	0
Haematological	0	1
Endocrine	0	2
Other	0	0

Overall	0	5

Data are number of participants (number of events). Participants could report more than one category of event.

**Table 5:** The presentation of percentage PANSS-derived responder rates (per-protocol set and intention-to-treat).

PANSS	Total *n*	<25% PANSS	25–49% PANSS	50–74% PANSS	75–100% PANSS
		Reduction *n* (%)	Reduction *n* (%)	Reduction *n* (%)	Reduction *n* (%)
PPS					
TJ-54 group	48	28 (58.3)	15 (31.3)	4 (8.3)	1 (2.1)
Placebo group	50	40 (80.0)	8 (16.0)	2 (4.0)	0 (0)
ITT					
TJ-54 group	54	29 (53.7)	18 (33.3)	6 (11.1)	1 (1.8)
Placebo group	60	44 (73.3)	12 (20.0)	4 (6.7)	0 (0)

There was no statistical significant difference. PPS: per-protocol set; ITT: intention-to-treat.
